# Phenology in the deep sea: seasonal and tidal feeding rhythms in a keystone octocoral

**DOI:** 10.1098/rspb.2022.1033

**Published:** 2022-10-26

**Authors:** Fanny Girard, Steven Y. Litvin, Alana Sherman, Paul McGill, Amanda Gannon, Christopher Lovera, Andrew DeVogelaere, Erica Burton, Dale Graves, Aaron Schnittger, Jim Barry

**Affiliations:** ^1^ Monterey Bay Aquarium Research Institute, 7700 Sandholdt Road, Moss Landing, CA 95039, USA; ^2^ Monterey Bay National Marine Sanctuary, National Ocean Service, National Oceanic and Atmospheric Administration, Monterey, CA 93940, USA

**Keywords:** cold-water coral, *Paragorgia arborea*, Sur Ridge, Monterey Bay National Marine Sanctuary, polyp activity, temporal dynamics

## Abstract

Biological rhythms are widely known in terrestrial and marine systems, where the behaviour or function of organisms may be tuned to environmental variation over periods from minutes to seasons or longer. Although well characterized in coastal environments, phenology remains poorly understood in the deep sea. Here we characterized intra-annual dynamics of feeding activity for the deep-sea octocoral *Paragorgia arborea*. Hourly changes in polyp activity were quantified using a time-lapse camera deployed for a year on Sur Ridge (1230 m depth; Northeast Pacific). The relationship between feeding and environmental variables, including surface primary production, temperature, acoustic backscatter, current speed and direction, was evaluated. Feeding activity was highly seasonal, with a dormancy period identified between January and early April, reflecting seasonal changes in food availability as suggested by primary production and acoustic backscatter data. Moreover, feeding varied with tides, which likely affected food delivery through cyclic oscillation in current speed and direction. This study provides the first evidence of behavioural rhythms in a coral species at depth greater than 1 km. Information on the feeding biology of this cosmopolitan deep-sea octocoral will contribute to a better understanding of how future environmental change may affect deep-sea coral communities and the ecosystem services they provide.

## 1. Background

How living organisms interact with their environment is a central question in ecology. Overall, environmental variation can have a profound impact on species from the individual to community levels [1,2]. In marine environments in particular, organisms have adapted to align with periodic environmental oscillations to optimize fitness [3,4]. However, although the influence of seasons and circadian solar or lunar rhythms on shallow-water ecosystems has been well characterized, it remains unclear for most of the deep sea (depth greater than 200 m), the largest ecosystem on the planet.

Traditionally, the deep sea had been considered a particularly stable environment where organisms cope with constant darkness, low temperature, slow currents and limited food [5]. In particular, with some exceptions (e.g. chemosynthetic ecosystems), deep-sea species primarily rely on food originating from surface primary production and subsequently transported to the seafloor through the biological pump (i.e. passive sinking of particle aggregates—decaying phytoplankton/fecal pellets; zooplankton migrations) [6]. Although estimating food availability in the deep sea can be challenging due to uncertainties regarding particulate organic carbon (POC) sinking rates and degradation processes, POC fluxes can vary episodically and seasonally, creating temporal structure at the greatest ocean depths [7,8].

Over the past decades, faunal behavioural rhythms in relation to day-night, tidal and seasonal cycles have been relatively well studied for mesopelagic [9,10] and upper-continental-slope benthic communities [11,12]. However, due to the technical and logistical challenges associated with obtaining high-resolution temporal data in the deep sea, limited information is available below the twilight zone (greater than 1000 m). Nevertheless, studies have shown that hydrodynamic and pressure changes related to tides, as well as seasonal dynamics, can also affect demersal fish and benthic communities in abyssal plains and deep-sea chemosynthetic ecosystems [13–16].

Technological advances and the emergence of deep-sea observatories have provided new opportunities for monitoring deep-sea ecosystems [17]. Monitoring represents a key tool in conservation science and is required to evaluate the efficacy of conservation measures (e.g. marine protected areas [18]) and the effects of anthropogenic activities or climate change [19,20]. Accordingly, there is a need for high-resolution temporal studies on deep-sea ecosystems requiring protection, such as ecologically or biologically significant marine areas (EBSAs), as defined by the Convention on Biological Diversity [21], and vulnerable marine ecosystems (VMEs; UNGA/FAO [22]).

Deep-sea coral ecosystems represent hotspots of abundance and diversity in the deep ocean [23] and play an important role in carbon cycling [24]. However, their generally high longevity and low growth rates make them particularly vulnerable to anthropogenic impacts [25,26]. For these reasons, deep-sea corals have been listed as EBSAs and VME indicators. Additionally, due to their ubiquity and life history, deep-sea corals have been defined as reliable biological indicators to detect environmental change in the deep ocean [27]. Despite their ecological importance and the multiple anthropogenic threats they are facing [28,29], temporal studies investigating the effect of environmental conditions on deep-sea corals remain limited.

Akin to other passive suspension feeders, resource acquisition represents an important factor constraining the health, dynamics and distribution of coral populations [30], particularly in the deep sea where food is limited [31]. While deep-sea corals often rely on fresh phytodetritus and partially degraded particulate organic matter (sinking from the surface or resuspended) [23], some species can adapt to changes in resource availability by opportunistically exploiting different food sources (i.e. zooplankton) [32–34]. However, to date, little information exists on the trophic ecology and phenology of most deep-sea coral species. In shallow water, studies have demonstrated an influence of seasonal and tidal cycles on coral feeding strategy through changes in currents, temperature, food quantity and quality [35–37]. Similarly, aquarium experiments and *in situ* observations of deep-sea corals (at depths of approximately 200 m) have demonstrated that their feeding behaviour varies diurnally and seasonally and is influenced by tidal currents (speed and direction) [12,38,39]. However, to date, no information is available for corals living in the aphotic deep sea (greater than 1000 m).

The goal of this study is to characterize temporal variation in feeding by the octocoral *Paragorgia arborea* (Linnaeus, 1758) in relation to environmental variability (i.e. currents, temperature and primary production) at a depth of 1230 m.

## 2. Methods

### (a) Study area and image acquisition

**Figure 1.**
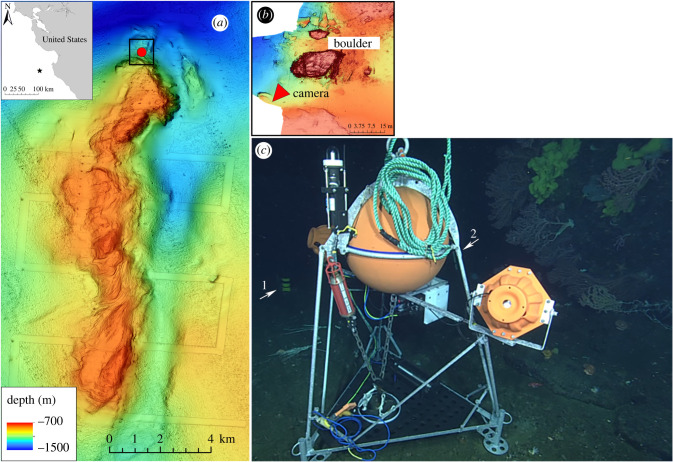
Study area and camera system. (*a*) Location of the study site (red dot) on Sur Ridge. The inset map indicates the location of Sur Ridge off Central California. (*b*) Study area and camera location. The red triangle shows the orientation of the time-lapse camera which was facing a large boulder hosting several species of deep-sea coral and sponges. (*c*) *In situ* photo of the camera system. The 300 kHz (1) and 1 MHz (2) ADCPs are visible in the background. (Online version in colour.)

Sur Ridge is a large rocky ridge located off the central California coast, within Monterey Bay National Marine Sanctuary (MBNMS; figure 1*a*). Since the discovery of dense and diverse deep-sea coral and sponge communities in 2013, Sur Ridge has been the focus of frequent oceanographic expeditions [40].

In March 2020, a time-lapse camera was deployed at a depth of 1230 m in the northern section of Sur Ridge (figure 1*a,b*). The camera system consisted of a digital still camera (Canon EOS 5D Mark IV) within a pressure housing mounted on a steel-framed lander (figure 1*c*). The system included two strobe lights mounted on both sides of the camera and a remotely releasable anchor weight permitting its return to the surface for recovery in March 2021.

The camera faced an approximately 10 m-high boulder hosting multiple coral and sponge species (figure 1*b*,*c*). With the exception of occasional failures between late September and early November (77 images lost), the camera captured one image every hour throughout the entire study period (6 March 2020–16 March 2021). One image per hour represented the highest achievable frequency to obtain one year of data with this camera system.

### (b) Image analysis

**Figure 2.**
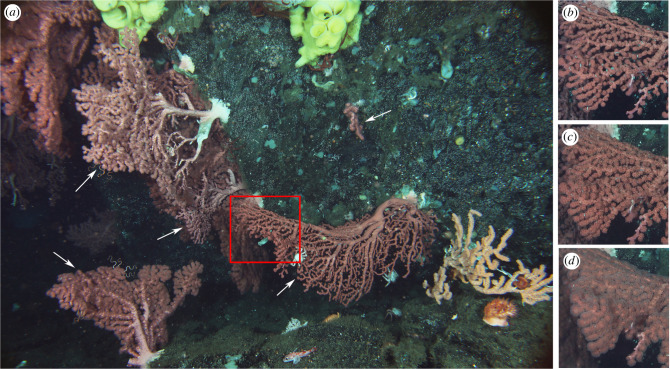
Image-based quantification of feeding activity. (*a*) Field of view of the time-lapse camera deployed on Sur Ridge between March 2020 and March 2021. Arrows point at the five *Paragorgia arborea* colonies annotated to quantify feeding activity. (*b–d*) Photographs representing coral branches (section of colony delineated by the red square) with retracted (closed; (*b*)), inflated (intermediate; (*c*)) and extruded (open; (*d*)) polyps. (Online version in colour.)

To quantify feeding activity, a total of 4439 images, corresponding to one image every 2 h, were annotated using the PAPARA(ZZ)I v. 2.8 software application [41]. Only half of the collected images were analysed (every 2 h instead of every hour) due to time constraints. Although two coral species were visible on the images, analyses focused on *Paragorgia arborea*, as only one colony of the bamboo coral *Keratoisis* sp. was present within the field of view (figure 2*a*).

For each image, the same five *P. arborea* colonies were classified into one of three categories: closed (retracted polyps; figure 2*b*), intermediate (polyps in intermediate state (inflated) and/or less than half of the colony with extruded polyps; figure 2*c*) and open (more than half of the colony with extruded polyps with fully extended tentacles; figure 2*d*). All polyps within a given colony were generally in the same state. However, when that was not the case, the proportion of the colony with open polyps was visually estimated and classified accordingly. The number of colonies in each of these states was then calculated for every image. The number of open colonies was used as proxy for feeding activity as polyps only feed when tentacles are extended in the water column.

### (c) Environmental characterization

To investigate the relationship between environmental conditions and coral feeding, two upward-looking Acoustic Doppler Current Profilers (ADCPs) with different working frequencies were deployed next to the time-lapse camera between March and December 2020 (figure 1*c*). The 1 MHz ADCP had a temporal resolution of 30 min and bin size of 50 cm, covering a depth range of 6 m, while the 300 kHz took measurements every 20 min over a range of 50 m with a bin size of 20 cm.

The two ADCPs measured bottom pressure, bottom temperature, acoustic backscatter, current speed and direction, eastward, northward and vertical flow speeds. Backscatter intensity averaged over all three beams was used as a proxy for suspended particle and zooplankton densities. Two different depth ranges were considered in the analyses: 1.5 to 2.5 m above bottom (mab; 1 MHz ADCP; referred as 2 m thereafter) representing the environmental conditions around corals and 12.5 to 13.5 mab (300 kHz ADCP; referred to as 13 m) representing the environmental conditions above the large boulder hosting the studied corals. This second depth range was included to evaluate the effect of the rock on bottom currents. For both ADCPs, current profiles were carefully examined using WinADCP v. 1.14 (300 kHz ADCP) and Surge v. 1.15.03 (1 MHz ADCP) software programs prior to depth selection to limit the impact of erroneous measurements. Because values from a single measurement can be noisy, measurements recorded over a distance of 1 m (corresponding to two and five bins for the 1 MHz and 300 kHz ADCPs, respectively) were averaged.

Additionally, a time series of net surface primary production (NPP) between March 2020 and March 2021, averaged within a 100 × 100 km area centred above Sur Ridge, was computed. NPP 8-day averages calculated using the Vertically Generalized Production Model algorithm, which is based on satellite-derived MODIS Chlorophyll-a concentration data, available light and temperature-dependent photosynthetic efficiency, were obtained from the Ocean Productivity website (http://sites.science.oregonstate.edu/ocean.productivity/; [42]).

### (d) Statistical analyses

To quantify seasonal variation in feeding activity, monthly differences in the number of colonies in each feeding category (open, intermediate and closed) were evaluated using generalized linear mixed models (GLMMs). Three separate models were tested, one per feeding category. Each time, the state of each of the five individual colonies monitored during the study period was coded as a binary variable (*e.g*. when testing for monthly differences in polyp extension: a value of 1 was assigned when polyps were open and 0 otherwise) and used as response variable in the model. A binomial distribution was thus used in all three models. To account for temporal autocorrelation and potential behavioural differences between colonies in this repeated measures design, date/time of observation and individual colony were included as random effects. Pairwise comparisons between months were then tested with Tukey's HSD using the *multcomp* R package (v.1.4.18). In addition, seasonal variations in the number of open colonies were represented in relation to current speed and direction using bivariate polar plots (*openair* v. 2.8.6 R package). All analyses were conducted in R [43].

Cross-correlation analyses between the number of open colonies and NPP were performed to investigate the relationship between surface primary productivity and coral feeding activity. To match the NPP dataset, numbers of open colonies were averaged over 8-day periods. Because the goal of this analysis was to determine whether NPP and feeding activity followed the same general trend, data were not detrended prior to cross-correlation analysis.

To identify cyclic variations and associated dominant periods in the different time series (number of open colonies and environmental variables measured by the ADCPs), Fast Fourier Transform periodograms were produced. As the temporal range and frequency of ADCP data acquisition differed from the time-lapse camera, a subset of the environmental time series that matched image time points (every 2 h) was used in the analyses. Moreover, because the ADCPs were deployed for a shorter time period (March–December 2020) than the camera (March 2020–March 2021), separate periodograms, one for each time period, were produced for feeding activity to allow for comparison. Feeding activity values missing due to camera failure (47 time points; see section 2a) were imputed by linear interpolation using the na_interpolation function of the *imputeTS* (v. 3.2) R package. Correlations between the number of open colonies and each environmental time series were then tested with cross-correlation analyses.

To identify which environmental variables had a significant effect on feeding activity, GLMMs with a binomial distribution were performed. Two separate models, one for each altitude (2 and 13 m) were tested. The feeding activity of each individual colony coded as a binary variable (1 when polyps were open and 0 otherwise) was used as response variable in the models. In all these GLMMs, bottom temperature, acoustic backscatter, eastward and northward flow speeds were included as fixed effects. Correlations between the different fixed effects were tested using Pearson's correlations prior to GLMM analysis. Because of its strong positive correlation with eastward flow, vertical flow speed was kept out of the models. Predictor variables were also standardized (by subtracting the mean and dividing by the s.d.) prior to analyses. To account for temporal autocorrelation, the effect of seasonality and colonies' individual behaviour, date and time of observation nested within month and individual colony were used as random effects in the GLMMs.

Finally, distance-based redundancy analyses (db-RDA) were performed to determine the proportion of the variance in feeding activity (considering the number of colonies in the three feeding categories) explained by environmental variables (bottom temperature, northward and eastward flow speeds, acoustic backscatter, NPP (8-day average and s.d.)) and time of the year (month of observation). To limit the effect of temporal autocorrelation, a randomly selected subset of the data was used in the analyses. Because time series of the number of open, intermediate and closed colonies were significantly autocorrelated up to a lag value of 10, the number of randomly selected observations included in the analyses corresponded to the total length of the time series divided by 10 (*n* = 273 and 287 for comparison with data collected by the 1 MHz and 300 kHz ADCPs, respectively). After comparing rank correlations between different dissimilarity indices, Gower's distance measure was used in the analyses. db-RDA analyses were performed with the R package *vegan* v. 2.5.7.

## 3. Results

### (a) Temporal dynamics of coral feeding activity

**Figure 3.**
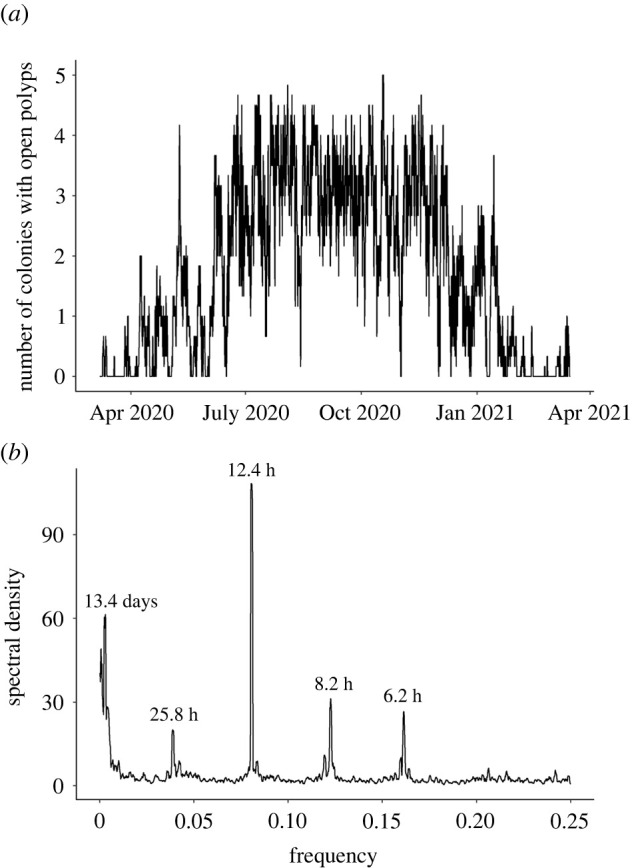
Temporal dynamics of feeding activity between March 2020 and March 2021. (*a*) Time series of the number of colonies with open polyps (feeding activity). The moving average over 12 h is represented. (*b*) Periodogram representing the dominant periods (1/frequency; unit = hours) in the feeding activity time series. Visible peaks represent the relative importance of the different frequencies in explaining oscillations in the time series.

The number of open *Paragorgia arborea* colonies, indicative of active feeding, followed a clear seasonal pattern between March 2020 and March 2021 (figure 3*a*). Specifically, feeding activity was highest between late June and late November, with peaks in early August and November. Conversely, feeding was minimal between late January and early April.

**Figure 4.**
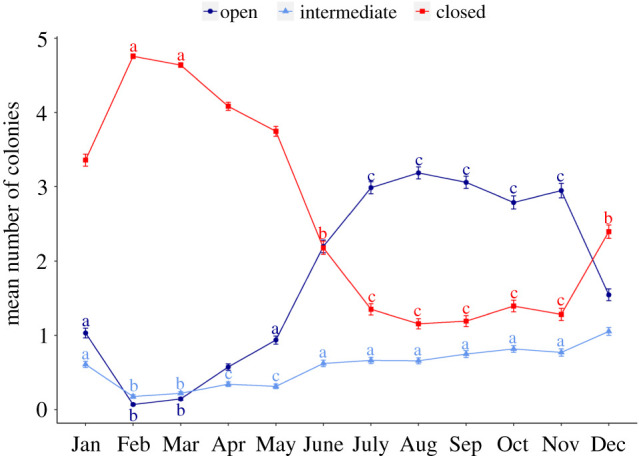
Mean (± s.e.) number of colonies with retracted (closed), inflated (intermediate) or extruded (open) polyps per month between March 2020 and March 2021. Letters represent statistical significance between months for each feeding category (colour codes) based on GLMM (alpha = 0.05, Tukey-adjusted): identical letters indicate equality within a given feeding category. (Online version in colour.)

Significant differences in the numbers of open, intermediate and closed colonies from month to month reflected this seasonality (figure 4). February and March were characterized by the highest number of closed colonies (means of 4.8 ± 0.03s.e. and 4.6 ± 0.03s.e., respectively) and lowest number of open colonies (means of 0.07 ± 0.01s.e. and 0.14 ± 0.02s.e., respectively). A major shift occurred in June, during which the mean number of open colonies significantly increased and exceeded that of closed colonies. Feeding activity then remained stable until November (between 2.8 ± 0.09s.e. and 3.0 ± 0.08s.e. open colonies, and 1.2 ± 0.08s.e. and 1.4 ± 0.07s.e. closed colonies, on average). Although relatively low (less than one colony per image on average), the number of intermediate colonies followed the same general trend as open colonies (figure 4).

Periodicities in feeding activity were identified from the periodogram, with five different peaks representing the dominant periods (1/frequency) of the time series (figure 3*b*). The number of open colonies appeared to vary with the lunar semi-diurnal tide (period of 12.4 h = M2 constituent/12.42 h) and, to a lesser extent, the moon phase (13.4 days approx. lunar fortnightly Mf/13.66 days), diurnal tide (25.8 h = O1/25.82 h) as well as other tidal constituents (8.2 = shallow water terdiurnal MK3/8.18; 6.2 = shallow water overtides of principal lunar constituent M4/ 6.21 h).

### (b) Relation between environmental conditions and coral feeding activity

**Figure 5.**
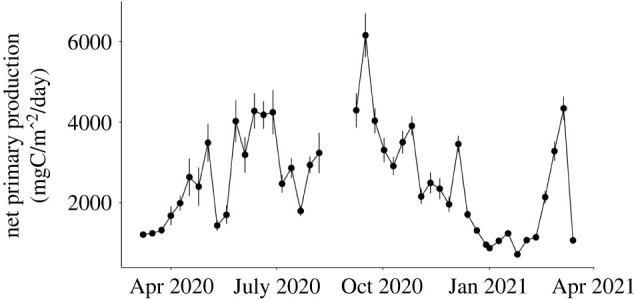
Time series of net surface primary production within a 100 × 100 km area centred above Sur Ridge between March 2020 and March 2021. Time points represent 8-day averages (± s.e.). Data were downloaded from the Ocean Productivity website (http://sites.science.oregonstate.edu/ocean.productivity/).

Overall, surface NPP averaged over 8-day periods showed a seasonal pattern, with an increase in NPP starting in early April and a peak in late September followed by a gradual decrease (figure 5). Cross-correlation analysis indicated a positive correlation between the 8-day average time series of the number of open colonies and surface NPP with the highest autocorrelation coefficient detected at lag 3 (lag of 24 days, ACF = 0.60 > critical value of 0.29).

At 13 mab, currents varied primarily along an east-northwest orientation throughout the year (electronic supplementary material, figure S1), with current speeds ranging from 0.16 to 120 cm s^−1^ and a mean of 17 cm s^−1^ ± 9.6s.d. Currents were generally weaker closer to the sea floor (2 mab), with speeds varying between 0.071 and 46 cm s^−1^ (average: 16 cm s^−1^ ± 8.8s.d.) along a dominant southeast-southwest orientation (electronic supplementary material, figure S1).

Time series of bottom temperature, acoustic backscatter and currents sampled at the same frequency as feeding activity were highly variable (March–December 2020; electronic supplementary material, figures S2 and S3) and displayed clear periodicities matching those identified for feeding activity (electronic supplementary material, figures S4 and S5). Bottom temperature, current speed and direction all matched semi-diurnal and diurnal tidal cycles regardless of depth. Additional tidal constituents (8.1 and 6.2 h periods) appeared to influence variations in dominant current speed and northward flow speed 2 mab (electronic supplementary material, figure S4). Periodic variation of several days was also detected for bottom temperature (32 days), current speed and northwest flow speed (19.7 days). Unlike the other environmental variables, acoustic backscatter density did not clearly vary over semi-diurnal to diurnal time scales, but exhibited spectral peaks matching spring/neap tidal oscillations (period of 14.8 days). Except for northwest flow speed (only semi-diurnal and diurnal signals detected 13 mab) and acoustic backscatter (8.2 h period detected 13 mab), environmental variables showed the same cyclic patterns at altitudes of 13 and 2 m.

Cross-correlation analyses revealed significant correlations between the number of open colonies and all environmental variables (electronic supplementary material, table S1). Specifically, feeding activity was positively correlated with bottom pressure (not recorded by 300 kHz ADCP), current speed and direction, and negatively correlated with bottom temperature, eastward and upward flow speeds recorded by both ADCPs. While feeding activity was positively correlated with acoustic backscatter at both altitudes, this correlation was only detected after a lag of 2 h at 13 mab. Conversely, the sign of the correlation between the number of open colonies and northwest flow speed differed depending on the altitude considered, with a positive correlation 13 mab and a negative one at 2 mab.

Similarly, GLMM results indicated a significant positive effect of acoustic backscatter density 2 mab on feeding activity, and a negative effect of bottom temperature, northward and eastward flow speeds (table 1). Results differed when considering environmental data collected 13 mab (table 1). In this model, only northward and eastward flow speeds had significant positive and negative effects on feeding activity, respectively.

**Table 1.** Coefficients of GLMMs testing the effect of environmental variables (bottom temperature, acoustic backscatter, northward and eastward flow speeds) collected by the two ADCPs 13 and 2 m above the seafloor between March and December 2020 on feeding activity. Data collection date and time nested within months as well as individual colony were included as random effects in the models to account for temporal autocorrelation and behavioural differences between colonies, respectively. Asterisks indicate significant results: *p-value < 0.05; **p-value < 0.01; ***p-value < 0.001.

fixed effects	13 mab		2 mab	
	estimate	s.e.	estimate	s.e.
intercept	−0.76	0.66	−0.74	0.65
bottom temperature	−0.045	0.036	−0.088*	0.036
northward flow speed	0.12**	0.042	−0.13***	0.035
eastward flow speed	−0.43***	0.041	−0.48***	0.035
acoustic backscatter	−0.005	0.042	0.25***	0.039

At altitudes of 2 and 13 m db-RDA was significant, indicating that environmental variables considered in this study explained 39 and 33%, respectively, of the variance in coral feeding activity (table 2; electronic supplementary material, figure S6). Except for bottom temperature and eastward flow speed at 13 mab, all variables were significant. At 2 mab, partial db-RDAs showed that the month of observation and average NPP explained most of the variance in feeding activity (33 and 17%, respectively), followed by NPP standard deviation and acoustic backscatter (both approx. 4–5%). However, currents explained less than 2% of the observed variance. While at 13 mab, northward flow speed and acoustic backscatter each explained about 6% of the variance in feeding activity; month of observation (29%) and average NPP (12%) remained the main explanatory variables (table 2; electronic supplementary material, figure S6).

**Table 2.** Results of db-RDA testing the relationship between coral feeding activity and environmental variables (bottom temperature, acoustic backscatter, northward and eastward flow speeds, net surface primary production and month). Adjusted *R*^2^ and significance levels of db-RDAs and partial db-RDAs are indicated. Data collected by the two ADCPs 13 and 2 m above the seafloor between March and December 2020 were analysed separately. *p-value < 0.05; **p-value < 0.01; ***p-value < 0.001.

environmental parameters	13 mab		2 mab	
	Adj. *R*^2^	significance	Adj. *R*^2^	significance
full model	0.33	***	0.39	***
bottom temperature	—	NS	—	NS
northward flow speed	0.064	***	0.010	*
eastward flow speed	—	NS	0.018	**
acoustic backscatter	0.067	***	0.042	***
average surface primary production	0.12	***	0.17	***
s.d. primary production	0.021	**	0.047	***
month	0.29	***	0.33	***

## 4. Discussion

Based on the analysis of a high-resolution time series of coral feeding activity measured over a year, this study is one of few documenting pronounced phenology in the deep sea. By linking the feeding behaviour of a deep-sea keystone species to tides and surface production, our results emphasize the strong connection between productive surface waters and the deep ocean.

### (a) Seasonality in coral feeding activity and food availability

Even at a depth of 1230 m, the feeding activity of the deep-sea coral *Paragorgia arborea* was highly seasonal, with a dormancy period characterized by low feeding activity lasting from late January to early April. Although relatively low throughout the study period, the number of colonies in an intermediate state followed the same general trend as open colonies, probably representing a transient state preceding feeding. Similar seasonal patterns in feeding activity have been documented for shallow-water benthic suspension feeders, and corals in particular, in temperate seas [3]. Periods of metabolic dormancy (low feeding and respiration rates) have been linked to low food availability during the oligotrophic season (winter or summer depending on geographic location [3,30,36]).

This relation between feeding at depth and food availability is reflected by the positive correlation detected between coral feeding activity and satellite-estimated NPP. Sur Ridge is part of the California Current System, characterized by a seasonal coastal upwelling [44,45]. In the spring and summer (generally from late March to late August), intensified equatorward winds along the coast drive the upwelling of cold, nutrient-rich waters, enhancing primary production at the surface. Between late August and November, upwelling progressively weakens but surface production remains relatively high until the upwelling stops around late December [44]. Overall, patterns in *P. arborea* feeding activity followed seasonal trends in surface primary production: winter dormancy corresponding to the period of minimal surface production, and the highest feeding activity occurring after June, typically the climax of the upwelling season [44], until November.

The seasonal increase in acoustic backscatter 13 and 2 mab, further supports this link between the surface and deep ocean. Whether they are periodic or seasonal, peaks in NPP can drive increased POC flux at depth [7,8]. However, depending on vertical transport mechanisms, POC sinking and remineralization rates may be highly variable [6]. Here, the lag of 24 days between surface primary production and coral feeding activity may correspond to the time required for surface-produced POC to sink to the sea floor, similar to sinking times reported for the region [7].

As illustrated by the association between polyp extension and higher acoustic backscatter density, food availability represents a key factor affecting coral feeding dynamics [37,46], and probably drove coral feeding over the study period.

### (b) Effect of food delivery on feeding activity

Over smaller temporal scales, variation in feeding activity followed clear cycles, with five dominant periods corresponding to different tidal constituents. As observed for other ecosystems in the northeast Pacific [15,47], temporal dynamics in coral feeding were dominated by the semi-diurnal tide. This is consistent with the observation that mixed diurnal/semi-diurnal tidal currents within the benthic boundary layer (BBL) off Central California are dominated by the M2 constituent [48]. Similarly, periodicities in current speed and direction time series matched diurnal and semi-diurnal tidal cycles, suggesting that tides modulate coral feeding through changes in current speed and direction. By driving food delivery, currents strongly influence the feeding behaviour of corals [49]. In general, polyps can only capture suspended particles within a limited, species-specific range of current speeds [50,51]. In particular, several deep-sea corals, including *Paragorgia arborea*, are preferentially located in areas of relatively high current speeds (i.e. mean greater than 20 cm s^−1^) [52]. Moreover, coral colonies are often oriented perpendicular to the prevailing current to optimize particle capture [53]. Accordingly, all *P. arborea* colonies visible on the time-lapse camera images were facing the dominant E-W current (figures 1 and 2).

Here, feeding was maximal during periods of strong westward flow (speed greater than 20 cm s^−1^) both at 13 and 2 mab (electronic supplementary material, figure S1). Depending on the altitude considered, northward flow affected polyp activity differently. Near the sea floor, higher feeding was associated with stronger southward flow, while the opposite was the case at an altitude of 13 m. This difference can be explained by the presence of the large (approx. 10 m high) boulder hosting the studied species, which likely diverts northward flow, emphasizing the importance of considering the impact of even small topographical features on local hydrodynamics when studying deep-sea coral ecosystems. Overall, the positive correlation between westward/southward flow speed and feeding activity could be due to a variety of factors related to turbulence regimes created by the local topography and/or colonies themselves, as well as the amount and quality of food brought by currents.

While dominated by the semi-diurnal tide, coral feeding activity also varied over lunar periods. Similarly, acoustic backscatter, a proxy for suspended particle and zooplankton, varied with spring/neap tidal oscillations. Interestingly, this association between spring/neap tides and particulate matter density within the BBL has been previously documented and attributed to the effect of varying turbulence on particle dynamics near the sea floor (deposition versus resuspension) [54]. Resuspended particulate organic matter may thus serve as an alternate food source to corals, especially during periods of low POC flux from surface waters [55].

### (c) Other factors potentially impacting polyp activity

The environmental variables measured in this study explained 33 to 39% of the variance in feeding activity suggesting that additional factors probably influence the feeding behaviour of *Paragorgia arborea*. The type and availability of food exploited by *P. arborea* at Sur Ridge are poorly known but likely have a strong influence on feeding behaviour. *P. arborea* is thought to feed primarily on fresh phytodetritus [56]; therefore, processes affecting sinking rates such as tidal pumping (rapid downwelling of surface waters) related to internal waves [57] could impact food quality and thus feeding activity. Moreover, *P. arborea* may opportunistically exploit other food sources depending on their availability. In particular, coral diets are known to change seasonally [34,58,59], and thus seasonal variation in regional phytoplankton [60,61] and zooplankton [62] communities are likely to impact the diet and feeding behaviour of *P. arborea*.

Even though polyp activity is primarily linked to food acquisition, it may be influenced by other factors. For instance, polyp expansion facilitates respiration by increasing the surface available for gas exchange [63]. In shallow temperate habitats, decreased polyp activity at high temperatures has been associated with reduced respiration [36,37,63]. While a similar negative correlation between temperature and polyp expansion has been identified in our study, temperatures were much lower and less variable (between 2.9 and 3.7°C) than in the aforementioned studies and the detected correlation may be an indirect consequence of tidal currents. Nonetheless, variation in oxygen concentrations at the study site may have affected respiration rates and thus polyp activity. In theory, the presence of corallivorous predators could also have affected polyp activity. However, such predators were rarely seen on coral colonies during the study period and no significant change in polyp behaviour was observed in the presence of predators (data on associated fauna, including predators, to be presented in a separate article).

Finally, part of the variability in feeding activity may be endogenously driven by phylogenetically conserved biological clocks. In shallow water, clock genes harmonizing biological rhythms with environmental cycles can provide a selective advantage to organisms by allowing them to better adapt to complex oscillatory environments (e.g. intertidal habitats) [64]. A regulation of activity rhythms by clock genes has been suggested for vent mussels in the deep sea [65] and shallow-water corals [66], but remains unknown for deep-sea corals.

### (d) Ecological implications

Our study indicates that the deep-sea octocoral *Paragorgia arborea* has adapted to optimize feeding under variable environmental conditions, likely maximizing its net rate of energy intake by primarily feeding when conditions are favourable (sufficient food availability and delivery). While the energetic cost of polyp extension is unknown, polyp activity has been associated with higher respiration rates in corals [63]. Therefore, lower metabolic activity during periods of low food availability (*e.g*. here in the winter) may be associated with low polyp activity. Additionally, corals with open polyps are likely more vulnerable as polyps could incur damage from predators or epibionts. Retracting polyps may thus be a way for corals to limit these risks. Overall, the identified phenology may be key in deep-sea environments where food is generally limited, optimizing energy acquisition and thus its allocation to growth and reproduction, directly impacting species fitness.

Moreover, active feeding has been associated with higher ecosystem productivity via increased mucus production by corals [33,67]. In addition to facilitating feeding and providing protection to corals, mucus can enhance carbon/nitrogen recycling, and ecosystem productivity as a whole, by fuelling microbial activity and the sponge loop [67,68]. In fact, although it has only been described for scleractinian corals, the sponge loop (sponges incorporate dissolved organic matter excreted by corals and produce detritus that can then be used by higher trophic levels) could play an important ecological role at Sur Ridge where dense coral and sponge assemblages co-occur. Cyclic changes in coral feeding activity may thus indirectly time the entire ecosystem.

Considering the link between surface productivity, hydrodynamic regimes and coral feeding at depth, our results suggest that future environmental changes could have far-reaching impacts on deep-sea coral ecosystems. In particular, climate change is projected to impact surface primary productivity and plankton communities [69] as well as deep-sea benthic ecosystems through warming, acidification and deoxygenation [70]. In the California Current System, a decrease in primary production and zooplankton biomass due to warming and increased stratification of surface waters have been projected [71]. As a result, corals may experience energy shortage and decreased fitness [30], possibly leading to the loss of the multiple ecosystem services they provide [24,72,73].

## 5. Conclusion

Overall, this study indicates that the lives of deep-sea organisms and the tempo of ecosystem function in the deep sea are likely more tightly tuned to key scales of environmental variation than previously recognized. Although the response of deep-sea organisms to seasonal changes in POC in deep-sea benthic ecosystems has been known for decades [8], their response to tidal rhythms in the deep sea is less well understood. However, our results suggest that tides could drive many of the processes shaping animal behaviour, often in ways we still know little about.

By linking polyp activity to surface productivity and tidally driven hydrodynamic changes, this study is the first to shed light on the influence of seasons and tides on the feeding behaviour of the deep-sea octocoral *Paragorgia arborea* at a depth greater than 1000 m, broadening our understanding of benthic-pelagic coupling processes in the deep sea. We provide important baseline information on the feeding biology of a cosmopolitan deep-sea octocoral, but the generality of these results for other deep-sea suspension feeders remains unknown. For example, the variation in the feeding activity of the single *Keratoisis* sp. colony visible in the images differed from *P. arborea*, and a larger sample size will be necessary to clearly characterize its feeding behaviour. Such information is essential to predict corals' response to future environmental change and evaluate the efficacy of conservation measures. Specifically, our results suggest that changes in food availability, for instance due to climate change, could translate early on into changes in coral feeding activity. Feeding activity could thus provide an important measure of the overall health of deep-sea coral ecosystems in a changing ocean.

## Data Availability

Feeding activity and environmental data: Dryad Digital Repository https://doi.org/10.5061/dryad.nvx0k6dw4 [74]. The data are provided in the electronic supplementary material [75].
